# Genetic predisposition in the 2′-5′A pathway in the development of type 1 diabetes: potential contribution to dysregulation of innate antiviral immunity

**DOI:** 10.1007/s00125-021-05469-5

**Published:** 2021-05-11

**Authors:** Kristina Pedersen, Martin Haupt-Jorgensen, Lars Krogvold, Simranjeet Kaur, Ivan C. Gerling, Flemming Pociot, Knut Dahl-Jørgensen, Karsten Buschard

**Affiliations:** 1 The Bartholin Institute, Department of Pathology, Rigshospitalet, Copenhagen, Denmark; 2 Division of Paediatric and Adolescent Medicine, Oslo University Hospital, Oslo, Norway; 3 Faculty of Dentistry, University of Oslo, Oslo, Norway; 4 Steno Diabetes Center Copenhagen, Gentofte, Denmark; 5 Department of Medicine, University of Tennessee, Memphis, TN USA; 6 Faculty of Health and Medical Sciences, University of Copenhagen, Copenhagen, Denmark; 7 Faculty of Medicine, University of Oslo, Oslo, Norway

**Keywords:** 2′-5′A pathway, Interferon α, 2′-5′ Oligoadenylate synthetase, Ribonuclease L, RNase L, Toll-like receptor 7, Type 1 diabetes, Type 1 interferon, Type 2 diabetes, Virus

## Abstract

**Aims/hypothesis:**

The incidence of type 1 diabetes is increasing more rapidly than can be explained by genetic drift. Viruses may play an important role in the disease, as they seem to activate the 2′-5′-linked oligoadenylate (2′-5′A) pathway of the innate antiviral immune system. Our aim was to investigate this possibility.

**Methods:**

Innate antiviral immune pathways were searched for type 1 diabetes-associated polymorphisms using genome-wide association study data. SNPs within ±250kb flanking regions of the transcription start site of 64 genes were examined. These pathways were also investigated for type 1 diabetes-associated RNA expression profiles using laser-dissected islets from two to five tissue sections per donor from the Diabetes Virus Detection (DiViD) study and the network of Pancreatic Organ Donors (nPOD).

**Results:**

We found 27 novel SNPs in genes nominally associated with type 1 diabetes. Three of those SNPs were located upstream of the 2′-5′A pathway, namely SNP rs4767000 (*p* = 1.03 × 10^−9^, OR 1.123), rs1034687 (*p* = 2.16 × 10^−7^, OR 0.869) and rs739744 (*p* = 1.03 × 10^−9^, OR 1.123). We also identified a large group of dysregulated islet genes in relation to type 1 diabetes, of which two were novel. The most aberrant genes were a group of IFN-stimulated genes. Of those, the following distinct pathways were targeted by the dysregulation (compared with the non-diabetic control group): *OAS1* increased by 111% (*p* < 1.00 × 10^−4^, 95% CI −0.43, −0.15); *MX1* increased by 142% (*p* < 1.00 × 10^−4^, 95% CI −0.52, −0.22); and *ISG15* increased by 197% (*p* = 2.00 × 10^−4^, 95% CI −0.68, −0.18).

**Conclusions/interpretation:**

We identified a genetic predisposition in the 2′-5′A pathway that potentially contributes to dysregulation of the innate antiviral immune system in type 1 diabetes. This study describes a potential role for the 2′-5′A pathway and other components of the innate antiviral immune system in beta cell autoimmunity.

**Graphical abstract:**

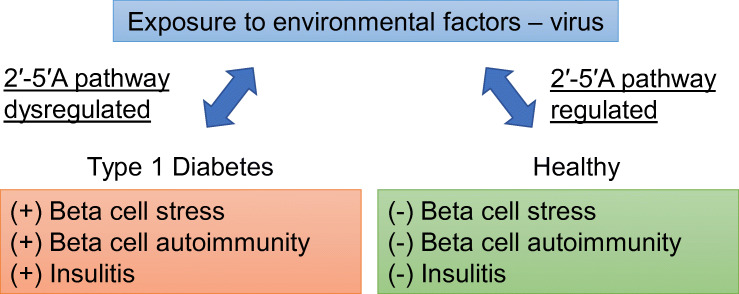

**Supplementary Information:**

The online version contains peer-reviewed but unedited supplementary material available at 10.1007/s00125-021-05469-5.

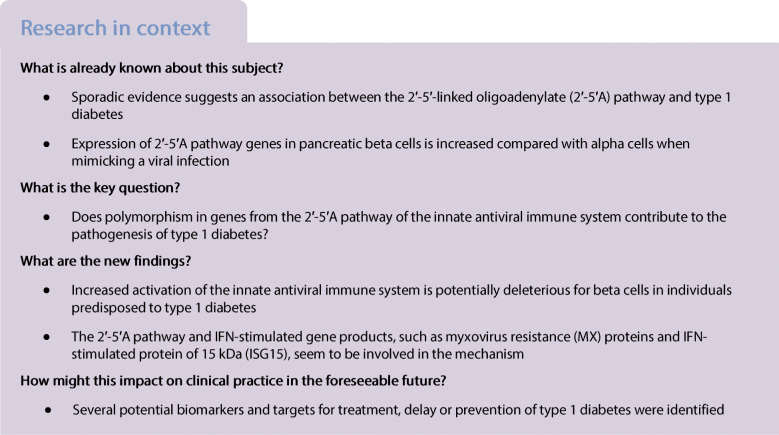



## Introduction

Type 1 diabetes is characterised by autoimmune T cell mediated destruction of pancreatic beta cells. Environmental factors may contribute to damage of beta cells and, particularly, viruses have been suspected to be implicated [1]. During a viral infection, the expression of type 1 IFN among the islet cells seems specific to beta cells [2]. Supporting the beta cell susceptibility hypothesis, it has been shown that treating alpha and beta TC3 cell lines with IFN-α and/or polyinosinic:polycytidylic acid [poly (I:C)], mimicking a viral infection, results in a six times higher 2′-5′ oligoadenylate synthetase (OAS) response in beta cells compared with alpha cells [3]. Why beta cells are more vulnerable than alpha cells to viral infection may be due to nitric oxide toxicity, antigen mimicry and/or possibly dysregulation of IFN-stimulated genes (ISGs) such as those in the 2′-5′-linked oligoadenylate (2′-5′A) pathway. The cellular tolerance to infection is known to be regulated by the innate antiviral immune response and varies among tissues and cell types [4]. This is especially problematic for beta cells due to their high metabolic activity resulting in vulnerability (e.g. during a viral infection).

In individuals with type 1 diabetes, the expression of IFN-α is increased in the islets of Langerhans and peripheral blood. Inflammation mediated by IFN signalling can potentially drive programmed cell death in beta cells and initiate autoreactivity against beta cell antigens [5]. Type 1 IFNs display differential tissue expression and binding affinity to their receptor complexes, which give rise to various outcomes including inflammation and antiviral, antiproliferative and immunomodulatory activity. Thus, type 1 IFNs and ISGs may be the link between genetics, the immune system, and environmental factors that have been shown to contribute to autoimmunity in type 1 diabetes [6].

Stimulation of pathogen-recognition receptors (PRRs) by pathogen-associated molecular patterns (PAMPs) induce the production of proinflammatory cytokines and type I IFN, which leads to transcription of ISGs. Some of the well-described ISGs include *ISG15*, *MX1*, *MX2*, *OAS1*, *OAS2*, *OAS3*, *OASL* and *RNASEL*. OAS/RNase L, IFN-stimulated protein of 15 kDa (ISG15), and Mx dynamin-like GTPase pathways show varying responsiveness to type 1 IFNs [7]. The 2′-5′A pathway, consisting of OAS proteins and the RNase L enzyme, is an RNA degradation pathway, which is induced by type 1 IFNs and activated by double-stranded RNA (dsRNA), an intermediate product of viral protein synthesis that is present in the cytosol of the cell. The activation of OAS by dsRNA results in polymerisation of ATP into 2′-5′A molecules. These 2′-5′A molecules activate latent RNase L, resulting in cleavage of single-stranded RNA (ssRNA) from viruses. However, RNase L can also inhibit protein synthesis of any other virus by cleaving host mRNA/ribosomal RNA, inhibiting translation. RNA cleaved by RNase L can then activate other cytoplasmic PRRs, resulting in further induction of type 1 IFN gene expression [8] and damage to the beta cell from within.

This study uses data from the genome-wide association study (GWAS) and islets transcriptomics from the Diabetes Virus Detection (DiViD) and the network of Pancreatic Organ Donors (nPOD) studies to investigate genetic variations and expression patterns in genes associated with innate antiviral immune pathways in relation to type 1 diabetes.

## Methods

### Tissue collection

Pancreas biopsies (*n*=5) were collected in the DiViD study as described previously [9]. Individuals with new-onset type 1 diabetes, 25–35 years of age, underwent a surgical minimal pancreatic tail resection by laparoscopy 3–9 weeks after diagnosis. The DiViD study was approved by the Norwegian Government’s Regional Ethics Committee (reference 2009/1907). Tissue from donors who were autoantibody-positive (*n*=12), had longstanding type 1 diabetes (*n*=20), had longstanding type 2 diabetes (*n*=8), or who were non-diabetic (control group; *n*=18) was used for RNA analysis and was acquired from nPOD with approval from the University of Tennessee Health Science Center local Institutional Review Board (reference 10-00848-XM).

### RNA analysis

RNA analysis was conducted on snap-frozen tissue obtained from the DiViD [9] and nPOD [10] tissue collections. Optimal cutting temperature embedded tissue slides were subjected to laser capture of islets conducted as previously described [11]. In brief, all islets in two to five tissue sections from each donor were captured, pooled, and RNA extracted using the Arcturus PicoPure RNA Isolation Kit (Applied Biosystems, Grand Island, NY, USA). Quality and quantity of RNA was determined on a Bioanalyzer 2100 instrument (Agilent Technologies, Santa Clara, CA, USA); RNA integrity numbers are shown in electronic supplementary material (ESM) Table 1. Samples with sufficient quantity and quality of RNA were then subjected to gene expression analysis using Affymetrix expression arrays (GeneChip Human Gene 2.0 ST; Thermo Fisher) and global scaling as normalising method as previously described [12].

### GWAS analysis

GWAS analysis included 64 genes that were researched for genetic associations with type 1 diabetes. Immunochip SNPs for type 1 diabetes were retrieved from Onengut-Gumuscu et al [13]. A cut-off *p* value <0.01 was used to retrieve nominally significant SNPs. SNPs within ±250 kb flanking regions of the transcription start site of the examined genes were identified. We used Encyclopedia of DNA Elements (ENCODE) regulatory features from the University of California Santa Cruz genome browser [14] (http://genome.ucsc.edu/) and RegulomeDB [15] to identify potential regulatory SNPs that were likely to affect the expression of the associated genes. We also integrated data from multiple expression quantitative trait locus (eQTL) studies [16] to identify SNPs associated with changes in expression (*cis*-eQTL) of the associated gene. The *cis*-eQTL effects were calculated using linear regression models in the selected tissues. Validated eQTLs from Westra et al [17] and GTEx2015_v6. GTEx2015_v6 eQTLs were computed using a ±1 Mb *cis* window around the transcription start site. Significance was determined using a *Q* value threshold. At least 70 samples per tissue are necessary to achieve the statistical power needed for this type of analysis. Predicted eQTL was calculated for pancreas and whole blood. Genotype–tissue expression predictions calculation were performed in tissues with at least ten samples. No *Q* value filtering was performed.

### Statistical analysis

Statistical analysis for RNA expression was performed using GraphPad Prism 8.0.2 (GraphPad, La Jolla, Ca, USA) and data are shown as mean ± SEM. Outliers were detected in each group using the ROUT method and a total of 21 datapoints across all genes and groups were identified and removed. All groups were tested for normal distribution by the D’Agostino–Pearson and Anderson Darling test. For comparison between groups, one-way ANOVA was used with Dunnett’s multiple comparison test and a 95% CI. A *p* value of <0.05 was considered significant (shown as *p < 0.05, **p < 0.01 and ***p < 0.001 in figures).

## Results

Selection of genes for analysis were based on their direct and/or indirect association upstream and downstream of the 2′-5′A pathway by examining the literature. We first conducted the GWAS study with 64 genes and, based on the resulting data, we selected 41 genes out of the original 64 for analysis of the RNA levels in islets from the nPOD and DiViD study. The individuals included in the GWAS study were all under the age of 17 years at diagnosis. In the nPOD study, the age at diagnosis was 3–26 years. The DiViD study participants were aged 24–35 years at diagnosis.

### SNPs in genes of the innate antiviral immune system show association to type 1 diabetes

GWAS identified 22 genes with 167 (158 unique) associated SNPs having *p* values <0.01 and ORs for the minor allele ranging from 0.5724 to 1.1841 (see ESM Table 2). Of these SNPs, 27 were nominally associated with type 1 diabetes. There were 15 genes that had at least one SNP with an eQTL *p* value <0.05 in either whole blood (validated eQLT) or pancreas (predicted eQTL). By comparing those 15 genes to the genes analysed for RNA expression in islets, seven genes (denoted by superscript ‘a’ in Tables 1 and 2) overlapped and were identified with significant type 1 diabetes SNPs with an eQTL signal.

**Table 1:** Total number of SNPs related to the innate antiviral immune system and associated with type 1 diabetes

Gene	Total no. of T1D SNPs (±250 kb)	No. of SNPs with eQTL signal	SNP most strongly associated with T1D	T1D SNP *p* value	OR (minor/major allele)
*ADAR*^a,b^	9	7	rs4845625	1.32 × 10^−3^	1.06/0.94
*CASP3*	2	1	rs17075783^b^	4.46 × 10^−3^	0.91/1.10
*CYBA*	4	2	rs4782429^b^	4.21 × 10^−3^	0.92/1.09
*EIF2AK2*	4	4	rs2247010^b^	3.73 × 10^−3^	0.94/1.06
*EIF3H*	1	1	rs1446534^b^	1.69 × 10^−4^	0.92/1.08
*GSPT1*	2	1	rs350234^b^	1.64 × 10^−2^	1.05/0.95
*IFIH1*^a^	90	50	rs2111485	3.81 ×10^−18^	0.85/1.18
*IFNG*^a^	4	3	rs11614178	1.55 × 10^−3^	0.96/1.05
*IL1R1*	8	6	rs4850992^b^	5.58 × 10^−3^	0.75/1.34
*IRF7*^a^	2	1	rs17758^b^	1.01 × 10^−2^	0.87/1.15
*MX2*^a,b^	2	1	rs7278439^b^	1.24 × 10^−3^	0.95/1.05
*NOD2*	4	4	rs2302759^b^	4.11 × 10^−3^	0.94/1.07
*OAS1*^a^	4	3	rs4767000^b^	1.03 × 10^−9^	1.12/0.89
*OAS3*^a^	3	2	rs4767000^b^	1.03 × 10^−9^	1.12/0.89
*TNFRSF1A*	15	3	rs10849451^b^	1.59 × 10^−3^	1.06/0.94

^a^Genes that also showed dysregulation on RNA expression level

^b^Newly discovered in association with type 1 diabetes

T1D, type 1 diabetes

**Table 2:** SNPs with an OR above 1.10 that were associated with type 1 diabetes

Gene	SNP details				Validated eQTL *p* value in whole blood	Predicted eQTL *p* value in pancreas	Predicted eQTL *p* value in whole blood
	SNP	*p* value (without *Q* value filtering)	OR (minor allele)	OR (major allele)			
*IFIH1*^a^	rs77088072^b^	9.94 × 10^−3^	0.793	1.261		1.40 × 10^−3^	4.30 × 10^−3^
	rs984971	2.40 × 10^−16^	0.849	1.178			2.50 × 10^−2^
	rs2068330	3.64 × 10^−16^	0.850	1.177			2.30 × 10^−2^
	rs3788964	3.99 × 10^−9^	0.858	1.165		2.80 × 10^−2^	
	rs1549020^b^	2.56 × 10^−12^	0.863	1.158			4.80 × 10^−3^
	rs3747517	4.98 × 10^−11^	0.871	1.148		3.50 × 10^−2^	2.20 × 10^−2^
	rs13422767	5.33 × 10^−8^	0.873	1.146		1.50 × 10^−2^	
	rs13023380	2.56 × 10^−12^	0.876	1.142			4.60 × 10^−2^
	rs7608315	9.56 × 10^−11^	0.884	1.131			2.50 × 10^−2^
	rs13022749^b^	1.60 × 10^−8^	0.893	1.120			5.10 × 10^−3^
*IFNAR1*^a^	rs2834136^b^	1.36 × 10^−2^	0.854	1.171			
*IFNG*^a^	rs7132610^b^	3.18 × 10^−3^	1.125	0.889			
*IL1R1*	rs79184888^b^	5.83 × 10^−3^	0.753	1.328			
	rs7565504^b^	6.70 × 10^−3^	0.754	1.326			
	rs6731416^b^	6.70 × 10^−3^	0.754	1.326			
	rs140672656^b^	6.33 × 10^−3^	0.784	1.275			
*IRF7*^a^	rs118067545^b^	8.48 × 10^−3^	0.836	1.196			
	rs3993797^b^	1.64 × 10^−2^	0.887	1.128			
*OAS1*^a^	rs1034687^b^	2.16 × 10^−7^	0.869	1.150	2.19 × 10^−11^		
	rs1015249^b^	1.25 × 10^−7^	0.895	1.117	2.7 × 10^−13^		
	rs739744^b^	1.03 × 10^−9^	1.123	0.890	1.11 × 10^−21^		
*OAS2*^a^	rs1034687^b^	2.16 × 10^−7^	0.869	1.150			
	rs4767000^b^	1.03 × 10^−9^	1.123	0.890			
	rs739744^b^	1.03 × 10^−9^	1.123	0.890			
*OAS3*^a^	rs1034687^b^	2.16 × 10^−7^	0.869	1.150		1.30 × 10^−2^	
	rs739744^b^	1.03 × 10^−9^	1.123	0.890	1.32 × 10^−4^		
*SP1*	rs17125267^b^	1.03 × 10^−2^	0.838	1.194			
	rs11170466	1.47 × 10^−5^	1.184	0.845			

The SNPs in this table were selected based on their eQTL *p* value in the specific tissues and their OR being above 1.10 for either the minor or the major allele

^a^Genes that also showed dysregulation on RNA expression level

^b^Newly discovered in association with type 1 diabetes

**Fig. 1:**
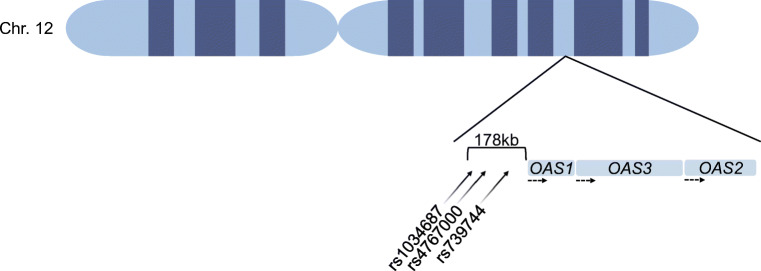
Overview of the OAS family of genes on chromosome 12 and the location of SNPs rs1034687, rs4767000 and rs739744

Four SNPs with a strong type 1 diabetes association and an eQTL signal were seen in *ADAR* (encoding RNA-specific adenosine deaminase), *IL1R1* (encoding IL-1 receptor type 1), *IRF7* (encoding IFN regulator 7) and the OAS gene family (Table 1). In *ADAR*, SNP rs4845625 (eQTL *p* = 1.32 × 10^−3^) had an OR of 1.06 for the minor allele and was located 135 kb downstream of the gene. In *IL1R1*, the novel SNP rs4850992 (eQTL *p* = 5.58 × 10^−3^) had an OR of 0.75 for the minor allele and was located 85 kb upstream of the gene. In *IRF7*, the novel SNP rs17758 (eQTL *p* = 1.01 × 10^−2^) had an OR of 0.87 for the minor allele and was located 30 kb upstream of the gene. SNP rs4767000 (eQTL *p* = 1.03 × 10^−9^) was located upstream of *OAS1* and *OAS3* with an OR of 1.12 for the minor allele. In *OAS2*, SNP rs4767000 (*p* value = 1.03 × 10^−9^, Table 2) also showed a significant *p* value with an OR of 1.12 for the minor allele but it did not have an eQTL signal. Three SNPs were located upstream of the OAS genes (Fig. 1). These SNPs were associated with and novel to type 1 diabetes as well as related to *OAS1*, *OAS2* and *OAS3*. In addition to the aforementioned rs4767000, the others were rs1034687 (*p* = 2.16 × 10^−7^, OR 0.869) and rs739744 (*p* = 1.03 × 10^−9^, OR 1.123).

As shown in Table 2, the gene encoding IFN induced with helicase domain 1 (*IFIH1*) had four SNPs with significant predicted eQTLs in the pancreas: rs77088072 (*p* = 9.94 × 10^−3^, OR 0.79); rs3788964 (*p* = 3.99 × 10^−9^, OR 0.86); rs3747517 (*p* = 4.98 × 10^−11^, OR 0.87); and rs13422767 (*p* = 5.33 × 10^−8^, OR 0.87). The SNP rs3788964 was located 35 kb upstream of the transcription start site in *IFIH1*, rs13422767 was located 24 kb downstream, and rs77088072 (a novel SNP in relation to type 1 diabetes) and rs3747517 were located inside the *IFIH1* gene. The SNP rs2111485 (Table 1) was located 14 kb downstream of *IFIH1* and was the SNP with the strongest eQTL signal associated with type 1 diabetes out of 90 SNPs found in this gene.

For *IL1R1*, rs4850992 (*p* = 5.58 × 10^−3^, OR 0.75) was located 87 kb upstream of the gene (Table 1). The SNP rs7565504 (*p* = 6.70 × 10^−3^, OR 0.75) was located 155 kb upstream of *IL1R1*, and the last SNP of interest in this gene was rs6731416 (*p* = 6.70 × 10^−3^, OR 0.75) located 160 kb upstream from transcription start site (Table 2). All three SNPs were to the best of our knowledge novel in relation to type 1 diabetes.

### Genes of the innate antiviral immune system show dysregulated RNA expression associated with type 1 diabetes

**Fig. 2:**
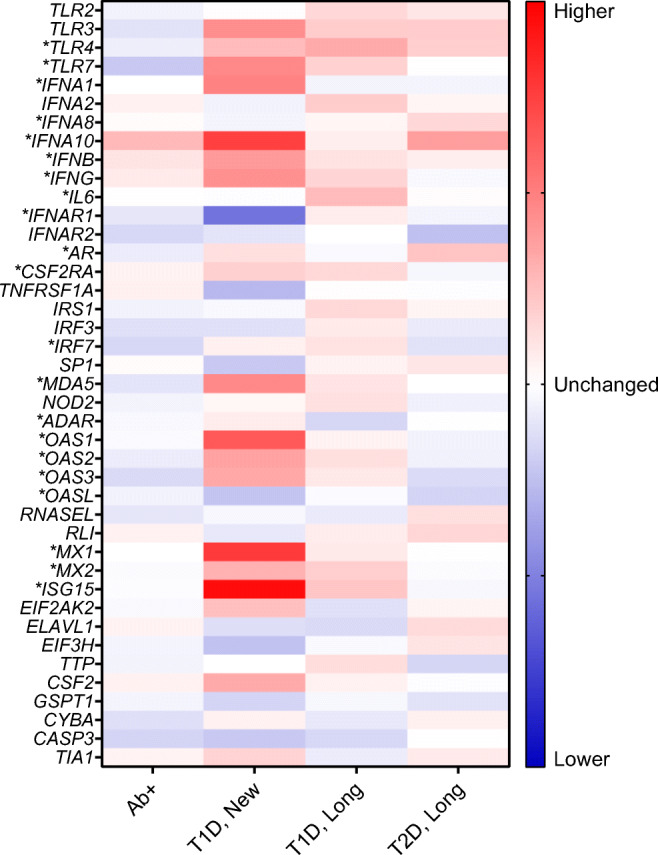
Heatmap of RNA expression in the analysed genes. Genes were normalised to the non-diabetic control group by calculating the ratio of the means in each group. Blue indicates lower RNA expression compared with the control group and red indicates higher RNA expression level. A total of 21 genes showed significantly (*) dysregulated RNA expression levels in either autoantibody-positive (Ab+), new-onset type 1 diabetes (T1D, New), longstanding type 1 diabetes (T1D, Long) or longstanding type 2 diabetes (T2D, Long) compared with controls

The heatmap revealed a strong dysregulation of most of the genes in individuals with new-onset type 1 diabetes from the DiViD study (Fig. 2). We selected 41 out of the 64 innate antiviral immune system genes analysed in the GWAS study for expression analysis in DiViD and nPOD islet tissue. Out of those 41 genes, two were novel in association with type 1 diabetes. Two genes showed lower RNA expression levels in autoantibody-positive individuals (*p* value <0.05). Thirteen genes showed either higher or lower expression levels in individuals with new-onset type 1 diabetes. Six genes showed higher expression levels in individuals with longstanding type 1 diabetes. Two genes showed higher expression levels and one gene was downregulated in longstanding type 1 diabetes. In general, the most aberrant genes were a group of IFGs.

### PAMP recognition and IFN induction are increased in new-onset type 1 diabetes

**Fig. 3:**
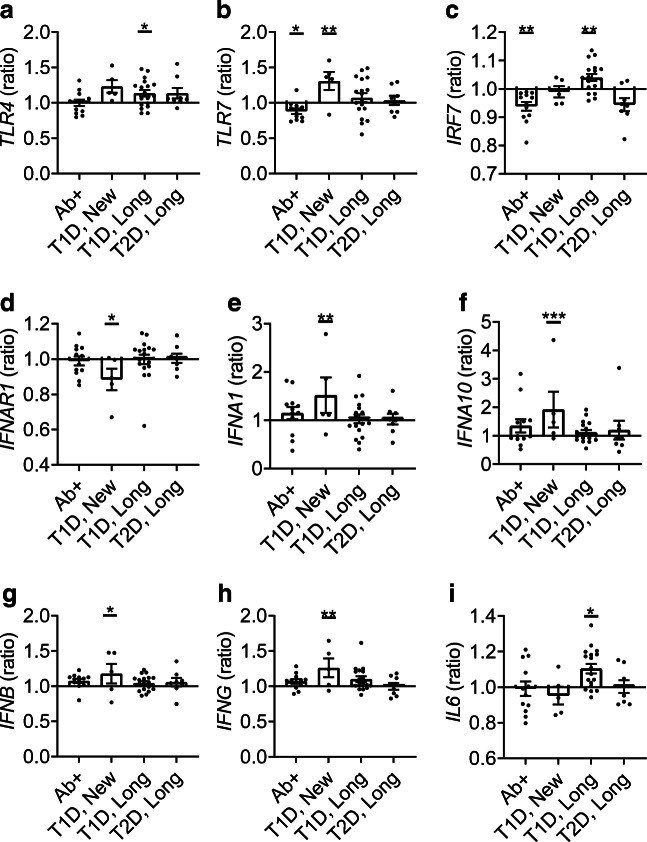
Genes involved in PAMP recognition and IFN induction. The RNA expression levels of *TLR4* (**a**), *TLR7* (**b**), *IRF7* (**c**), *IFNAR1* (**d**), *IFNA1* (**e**), *IFNA10* (**f**), *IFNB* (**g**), *IFNG* (**h**) and *IL6* (**i**) were transformed, and a mean was calculated. The ratio between the mean expression level in each group (autoantibody-positive [Ab+], new-onset type 1 diabetes [T1D, New], longstanding type 1 diabetes [T1D, Long], longstanding type 2 diabetes [T2D, Long]) and the non-diabetic control group was graphed. *p < 0.05, **p < 0.01 and ***p < 0.001 vs control group

Expression of the nine genes involved in the initial innate antiviral immune response is shown in Fig. 3. The expression of the gene encoding toll-like receptor 7 (*TLR7*) was significantly increased by 70% in individuals with new-onset type 1 diabetes compared with the non-diabetic control group (*p* = 9.60 × 10^−3^, 95% CI −0.42, −0.05, Fig. 3b). *IFNA1* expression was increased by 75% (*p* = 1.30 × 10^−3^, 95% CI −0.44, −0.09, Fig. 3e) and *IFNA10* expression was increased by 136% (*p* < 1.00 × 10^−4^, 95% CI −0.50, −0.18, Fig. 3f) in individuals with new-onset type 1 diabetes. Expression of *IFNB* (*p* = 4.00 × 10^−2^, 95% CI −0.30, −0.006, Fig. 3g) and *IFNG* (*p* = 2.30 × 10^−3^, 95% CI −0.32, −0.06, Fig. 3h) was increased by 58% and 65%, respectively, in new-onset type 1 diabetes. Interestingly, in the same group, expression of the gene encoding IFN-α and -β receptor subunit 1 (*IFNAR1*) was decreased by 47% (*p* = 1.80 × 10^−2^, 95% CI −0.04, −0.05, Fig. 3d).

In individuals with longstanding type 1 diabetes, gene expression level was increased by 47% for *TLR4* (*p* = 2.90 × 10^−2^, 95% CI −0.29, −0.01, Fig. 3a) and 36% for *IL6* (*p* = 1.30 × 10^−2^, 95% CI −0.18, −0.02, Fig. 3i). *IRF7* expression was decreased by 17% in autoantibody-positive individuals (*p* = 6.10 × 10^−3^, 95% CI 0.02, 0.14, Fig. 3c); the expression of this gene was increased in longstanding type 1 diabetes by 14% (*p* = 4.00 × 10^−2^, 95% CI −0.11, −0.002, Fig. 3c).

### Multiple ISGs are highly elevated in new-onset type 1 diabetes

**Fig. 4:**
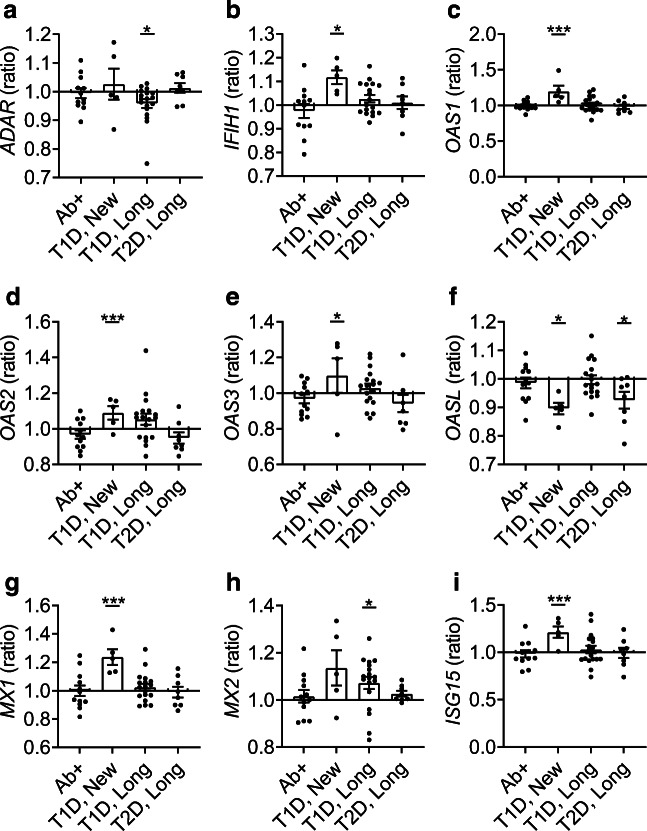
Cytokine genes and ISGs that were upregulated by IFN induction. The RNA expression levels of *ADAR* (**a**), *IFIH1* (**b**), *OAS1* (**c**), *OAS2* (**d**), *OAS3* (**e**), *OASL* (**f**), *MX1* (**g**), *MX2* (**h**) and *ISG15* (**i**) were transformed, and a mean was calculated. The ratio between the mean expression level in each group (autoantibody-positive [Ab+], new-onset type 1 diabetes [T1D, New], longstanding type 1 diabetes [T1D, Long], longstanding type 2 diabetes [T2D, Long]) and the non-diabetic control group was graphed. *p < 0.05 and ***p < 0.001 vs control group

In Fig. 4, the nine genes shown are upregulated or downregulated by the genes (shown in Fig. 3) involved in the initial response of the innate antiviral immune pathway. In individuals with new-onset type 1 diabetes, the expression of *OAS1* was increased by 111% (*p* < 1.00 × 10^−4^, 95% CI −0.43, −0.15, Fig. 4c), the expression of *MX1* was increased by 142% (*p* < 1.00 × 10^−4^, 95% CI −0.52, −0.22, Fig. 4g) and the expression of *ISG15* was increased by 197% (*p* = 2.00 × 10^−4^, 95% CI −0.68, −0.18, Fig. 4i) compared with non-diabetic control individuals. *OASL* expression was decreased by 23% (*p* = 2.40 × 10^−2^, 95% CI −0.06, −0.08) in individuals with new-onset type 1 diabetes (Fig. 4f). *MX2* expression was increased by 24% in individuals with longstanding type 1 diabetes (*p* = 3.80 × 10^−2^, 95% CI −0.18, −0.004, Fig. 4h). Moreover, *ADAR* expression was decreased by 17% in individuals with longstanding type 1 diabetes (*p* = 4.60 × 10^−2^, 95% CI 0.001, 0.2, Fig. 4a).

## Discussion

Our study is the first to investigate all major innate antiviral immune response pathways by combining analysis of GWAS data and analysis of RNA expression levels in islets from the DiViD and nPOD study populations. The GWAS study identified 168 type 1 diabetes-associated SNPs (159 unique SNPs), of which 27 were novel in relation to type 1 diabetes and are predicted to affect the expression of the associated genes. The islet RNA expression analysis identified two genes (*ADAR* and *MX2*) that were novel in having a direct relation to type 1 diabetes, out of 19 dysregulated genes. The highest degree of dysregulation (13 genes) was seen in individuals with new-onset type 1 diabetes compared with non-diabetic individuals. In autoantibody-positive individuals two genes were dysregulated in those with longstanding type 1 diabetes, six genes were dysregulated and in those with type 2 diabetes, three genes were dysregulated at the mRNA level. We found multiple ISGs, such as the whole OAS family of genes, that were aberrant in individuals with type 1 diabetes and these genes had several SNPs associated with the disease. Another interesting result was found in genes upstream of the 2′-5′A pathway, such as those encoding type 1 IFN and toll-like receptors.

The gene with the highest number of SNPs in our study was *IFIH1*, which is also referred to as *MDA5* (encoding melanoma differentiation-associated 5 [MDA5] protein). This gene, a cytosol PRR that recognises dsRNA and prevents early replication of viruses, had 50 SNPs predicted to affect its expression. Several SNPs in *IFIH1*, which could be linked to risk of and protection against type 1 diabetes, have previously been identified and may modify the structure and function of *IFIH1* [18].

The toll-like receptor genes and *IRF7* are important in the induction of type 1 IFN and upregulation of ISGs, especially the 2′-5′A pathway, and for further immune response through PRRs such as MDA5 [19]. Tight regulation of *IRF7* expression and activity is imperative for appropriate type 1 IFN production [20]. A total of three SNPs were found in *IRF7* and they could interfere with *IRF7* expression or its ability to bind a promoter region.

*IFNAR1* was downregulated in individuals with new-onset type 1 diabetes and it had an SNP associated with type 1 diabetes. Moreover, several *IFNA* isoforms, as well as *IFNB*, were upregulated. IFN-α and IFN-β share the IFN-α and -β receptor subunit 1 as a common cell surface receptor. Interestingly, the receptor for the two cytokines, IFN-α and IFN-β, being downregulated but the cytokines themselves being upregulated could result in inflammation and destruction of beta cells in the islets of individuals with type 1 diabetes.

Beta cells are especially vulnerable to infection, potentially through dysregulation of the innate antiviral immune system leading to inflammation and destruction [2]. Dysregulation of IFN-α or the 2′-5′A pathway caused by either a genetic predisposition or environmental factors has been associated with type 1 diabetes in different ways. Moreover, studies mimicking a viral response by poly (I:C) or type 1 IFN have shown that RNase L increases the expression of proinflammatory genes in the pancreas [21] and this might also contribute to the pathogenesis of type 1 diabetes.

The OAS genes were highly overexpressed in islets of individuals with new-onset type 1 diabetes and had several eQTL SNPs. In these individuals, *OAS1*, *OAS2* and *OAS3* were all upregulated whereas the *OASL* gene, which has an OAS-like domain but lacks the synthetase activity, was downregulated. We found three novel SNPs within the promoter region of *OAS1*, *OAS2* and *OAS3* that could potentially affect the expression of the OAS family of genes. High levels of *OAS1*, *OAS2* and *OAS3* indicate an antiviral response or a higher basal protein level in the cell. Whether *OAS1*, *OAS2* and *OAS3* are able to properly activate RNase L [22] or other downstream proteins such as retinoic acid-inducible I or MDA5 is crucial for antiviral responses. Therefore, dysregulation of *OAS1*, *OAS2*, *OAS3* and *OASL* could indicate lack of the antiviral response and/or a higher degree of virus replication in the cell [23]. In addition, other studies have shown that the 2′-5′A pathway could potentially regulate expression of ISG15 [24], activate MDA5 and even influence IFN expression [8]. The OAS/RNase L activation has also been associated with programmed cell death even without the presence of a pathogen [25].

We found that *MX1* was upregulated in new-onset type 1 diabetes and that *MX2* was upregulated in longstanding type 1 diabetes as well as having two eQTL SNPs. Both *MX1* and *MX2*, members of a gene family encoding dynamin-like large GTPases, suppress a wide but different variety of virus. Despite the similarities in sequence and structure, MX1 and MX2 have different mechanisms of action against viruses as well as which type of viruses they eliminate [26]. To the best of our knowledge, there is no previous evidence of a direct association between *MX1* and *MX2* and type 1 diabetes. Hence, this finding is novel and could be central for understanding virus-induced type 1 diabetes.

The majority of ISGs were upregulated in individuals with new-onset type 1 diabetes; however, in longstanding type 1 diabetes *ADAR* was downregulated and *MX2* was upregulated (both ISGs). The protein encoded by *ADAR* has a dual role as it either modifies or regulates the innate antiviral immune response. It can prevent an unwanted MDA5 and type 1 IFN feedback loop by preventing MDA5 and OAS activation through A-to-I editing of dsRNA. However, if *ADAR* is not regulated correctly, the A-to-I editing activity could either prevent or create an overactivation of an antiviral immune response.

Regarding type 2 diabetes, we only found three dysregulated genes at the mRNA level when compared with healthy control individuals, indicating that viral infection is not likely to contribute to this disease. *OASL* was downregulated and has shown effects on C-reactive protein, γ glutamyltransferase and LDL-cholesterol, which are all related to cardiovascular events that are frequent in individuals with type 2 diabetes as well as inflammation [27].

Lundberg et al investigated the RNA expression of several innate antiviral immune system genes in laser-dissected islet tissue from the DiViD study, of which 20 samples were also analysed in our study. However, nPOD islet tissue and GWAS data were not included in this study, and a smaller control group (*n*=5) was used [28]. As in our study, Lundberg et al [28] found the following genes to be significantly elevated: *IFIH1*; *MX1*; *IL6*; *ISG15*; and *OAS1*. This verifies an involvement of the innate antiviral immune response in new-onset type 1 diabetes.

A limitation of this study is the age difference between the individuals included in the GWAS study and the RNA expression study. It is well known that in type 1 diabetes there are different endotypes, which can be defined according to age [29, 30]. Thus, an endotype can be defined as those individuals diagnosed before the age of 7 years, those diagnosed after the age of 13 years, and those diagnosed between 7 and 12 years of age that can belong to either of the two endotypes. The pathogenesis of an endotype is defined by the type of infiltrating immune cells causing insulitis and by how much proinsulin and insulin are colocalised in beta cells [29]. In the current study, a direct association between the GWAS, the nPOD and DiViD cohorts should only be stated with caution, as the participants are from different endotypes. It is known that genetic variants in type 1 diabetes, typically located near genes and acting both in pancreatic beta cells and immune cells, are more prevalent in the endotypes diagnosed before the age of 7 years [30]. We were not able to correct for age, sex, BMI, HLA and/or autoantibody status due to small sample sizes; however, all this information can be found in ESM Table 1. This information can be combined with the information shown in ESM Figs 1 and 2, wherein each data point from the nPOD and DiViD analysis has an identifier correlating with information shown in ESM Table 1.

**Fig. 5:**
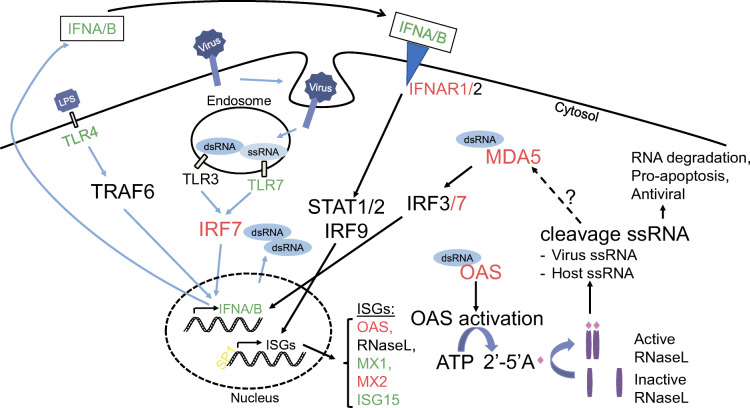
The activation and the feedback loop of the innate antiviral immune system during a viral infection. The RNA dysregulated genes/proteins where SNPs were also found in the GWAS study are highlighted in red. Genes/proteins that were only dysregulated at the RNA level are highlighted in green. *SP1* is highlighted in yellow because we only found SNPs in this gene associated with type 1 diabetes. The figure illustrates the multitude of genes involved in the potential overactivation in response to an infection in type 1 diabetes. LPS, lipopolysaccharide

In conclusion, innate antiviral immune sensors capable of detecting and clearing ssRNA, were downregulated in autoantibody-positive individuals and upregulated in individuals with new-onset type 1 diabetes. These genes included *TLR7*, *OAS*, *MX1*, multiple type 1 IFN genes and *IFIH1* (Fig. 5). In conjunction, we found polymorphisms in *OAS*, *MX1* and *IFIH1* that indicate predisposition to type 1 diabetes. The DiViD study previously found footprints of virus in islets from individuals with type 1 diabetes [31]. Thus, we hypothesise that environmental factors (e.g. virus) in predisposed and/or autoantibody-positive individuals stimulate dysregulated 2′-5′ pathway through toll-like receptor 7 and type 1 IFN in the innate immune system, causing inflammation in the islets and progression to diabetes. Identification and avoidance of diabetogenic viruses that can stimulate the toll-like receptor 7 pathway in early disease development may prevent or delay type 1 diabetes. It is our hope that further research on the role of toll-like receptors, the 2′-5′A pathway and other ISGs will lead to better prognostic markers and new therapeutic drugs.

## Supplementary Information

ESM (PDF 297 kb)

## Data Availability

All data generated and analysed during this study are included in this published article and its ESM. Raw data are published online (https://www.dropbox.com/s/egshju5x6d4j0or/GWAS%20study.xlsx?dl=0;https://www.dropbox.com/s/1r7lrxba1fb0atk/nPOD%20and%20DiViD%20studies.csv?dl=0).

## Abbreviations

2′-5′A: 2′-5′-Linked oligoadenylate
DiViD: Diabetes Virus Detection
dsRNA: Double-stranded RNA
eQTL: Expression quantitative trait locus
GWAS: Genome-wide association study
ISG: IFN-stimulated gene
MDA5: Melanoma differentiation-associated 5
nPOD: Network of Pancreatic Organ Donors
OAS: 2′-5′ Oligoadenylate synthetase
PAMP: Pathogen-associated molecular pattern
Poly (I:C): Polyinosinic:polycytidylic acid
PRR: Pathogen-recognition receptor
ssRNA: Single-stranded RNA
