# Supplementary material for: Genetic predisposition in the 2′-5′A pathway in the development of type 1 diabetes: potential contribution to dysregulation of innate antiviral immunity
Source: Diabetologia. 2021 May 11;64(8):1805–15. doi: 10.1007/s00125-021-05469-5 (PMC8245375; doi:10.1007/s00125-021-05469-5)
Supplement: Supplementary file 1 — (PDF 297 kb) [file 125_2021_5469_MOESM1_ESM.pdf]

ESM Table 1 Overview of the demographic features, HLA, autoantibody status, RNA integrity number (RIN) in all the individuals and patients used for the mRNA expression analysis from nPOD and DiViD studies.

Demographic features of the patients

	Patient code	Age (years)	Sex	Disease	Disease duration (years)	Source	BMI	RIN	Age at diagnosis (years)
1	IG1_6013-04PT(C)	65	Male	Control	0	nPOD	24.2	5.1	-
2	IG2_6024-01PT(C)	21	Male	Control	0	nPOD	27.8	4.2	-
3	IG3_6048-04PT(C)	30	Male	Control	0	nPOD	20.6	4.6	-
4	IG4_6075-06PB(C)	16	Male	Control	0	nPOD	14.9	6.9	-
5	IG5_6012-02PT(C)	68	Female	Control	0	nPOD	23.7	7.9	-
6	IG6_6099-02PB(C)	14.2	Male	Control	0	nPOD	30	5.2	-
7	IG7_6140-04PT(C)	38	Male	Control	0	nPOD	21.7	5.6	-
8	IG8_6162-01PT(C)	22.7	Male	Control	0	nPOD	28.9	4.1	-
9	IG9_6168-04PT(C)	51	Male	Control	0	nPOD	25.2	6.8	-
10	IG10_6227-02PT(C)	17	Female	Control	0	nPOD	26.4	6	-
11	IG11_6129-02PT(C)	42.9	Female	Control	0	nPOD	23.4	5.1	-
12	IG12_6165-04PT(C)	45.8	Female	Control	0	nPOD	25	5.5	-
13	IG13_6102-04PT(C)	45.1	Female	Control	0	nPOD	35.1	6.9	-
14	IG14_6229-02PT(C)	31	Female	Control	0	nPOD	26.9	6.6	-
15	IG15_6251-02PT(C)	33	Female	Control	0	nPOD	29.5	6.8	-
16	IG16_6010-04PT(C)	47	Female	Control	0	nPOD	19.7	6.5	-
17	IG17_6179-04B PB(C)	20	Female	Control	0	nPOD	20.7	7	-
18	IG18_6019-04PT(C)	42	Male	Control	0	nPOD	31	4.8	-
1	IG40_6080-01PT(AB)	69.2	Female	Ab+	0	nPOD	21.3	6.2	-
2	IG41_6123-02PT(AB)	23.2	Female	Ab+	0	nPOD	17.6	4.9	-
3	IG42_6158-10PT(AB)	40.3	Male	Ab+	0	nPOD	29.7	5.1	-
4	IG43_6167-08PT(AB)	37	Male	Ab+	0	nPOD	26.3	4.7	-
5	IG44_6171-01PT(AB)	4.4	Female	Ab+	0	nPOD	14.8	4.4	-
6	IG45_6044-02PT(AB)	41.4	Male	Ab+	0	nPOD	27.4	4.1	-
7	IG46_6101-04PT(AB)	64.8	Male	Ab+	0	nPOD	34.3	5.8	-
8	IG47_6154-06PT(AB)	48.5	Female	Ab+	0	nPOD	24.5	5.8	-
9	IG48_6156-06PT(AB)	40	Male	Ab+	0	nPOD	19.8	6.8	-
10	IG49_6181-06APT(AB)	31.9	Male	Ab+	0	nPOD	21.9	4.5	-

11	IG50_6197-08PB(AB)	22	Male	Ab+	0	nPOD	28.2	6.6	-
12	IG51_6147-02PB(AB)	23.8	Female	Ab+	0	nPOD	32.9	5.5	-
1	IG64_DiViD-6(Dg)	35	Male	New T1D	0.1	DiViD	26.7	4	35
2	IG65_DiViD-5(Dg)	24	Female	New T1D	0.1	DiViD	28.6	3.5	24
3	IG66_DiViD-4(Dg)	31	Male	New T1D	0.1	DiViD	25.6	4	31
4	IG67_DiViD-3(Dg)	34	Female	New T1D	0.2	DiViD	23.7	7.3	34
5	IG68_DiViD-2(Dg)	24	Male	New T1D	0.1	DiViD	20.9	5.7	24
1	IG20_6070-06PT(D1)	22.6	Female	T1D	7	nPOD	21.6	5.2	15.6
2	IG21_6084-02PT(D1)	14.2	Male	T1D	4	nPOD	26.3	4.2	10.2
3	IG22_6088-02PH(D1)	31.2	Male	T1D	5	nPOD	27	6.2	26.2
4	IG23_6180-04PT(D1)	27.1	Male	T1D	11	nPOD	25.9	4.3	16.1
5	IG24_6224-01PT(D1)	21	Female	T1D	1.5	nPOD	22.8	6	19.5
6	IG25_6243-02PT(D1)	13	Male	T1D	5	nPOD	21.3	3.7	8
7	IG26_6228-04PT(D1)	13	Male	T1D	0	nPOD	17.4	6.2	13
8	IG27_6209-01PT(D1)	5	Female	T1D	0.3	nPOD	11.95	3.8	4.8
9	IG28_6038-06PH(D1)	37.2	Female	T1D	20	nPOD	30.9	4.1	17.2
10	IG29_6046-03PT(D1)	18.8	Female	T1D	8	nPOD	25.2	5	10.8
11	IG30_6069-06PT(D1)	22.9	Male	T1D	7	nPOD	28.8	5	15.9
12	IG31_6195-06PT(D1)	19.2	Male	T1D	5	nPOD	23.7	5.1	14.2
13	IG32_6052-03PB(D1)	12	Male	T1D	1	nPOD	20.3	5	11
14	IG33_6268-02PT(D1)	12	Female	T1D	3	nPOD	26.6	6.1	9
15	IG34_6265-02PH(D1)	11	Male	T1D	8	nPOD	12.9	3.9	3
16	IG35_6196-04PT(D1)	26	Female	T1D	15	nPOD	26.6	7.8	11
17	IG36_6211-04PT(D1)	24	Female	T1D	4	nPOD	24.4	4.7	20
18	IG37_6113-01PH(D1)	13.1	Female	T1D	1.6	nPOD	24.8	4.9	11.5
19	IG38_6264-02PB(D1)	12	Female	T1D	9	nPOD	22	5.4	3
20	IG39_6135-01PT(D1)	43.5	Male	T1D	21	nPOD	28.7	7.4	22.5
1	IG56_6188-06PT(D2)	36.1	Male	T2D	0	nPOD	30.6	6.1	36.1
2	IG57_6114-03PT(D2)	42.8	Male	T2D	2	nPOD	31	6.6	40.8
3	IG58_6249-01PT(D2)	45	Female	T2D	15	nPOD	32.3	7.4	30
4	IG59_6275-02PT(D2)	48	Male	T2D	2	nPOD	41	6.4	46
5	IG60_6273-04PT(D2)	45	Female	T2D	2	nPOD	39.1	6.1	43
6	IG61_6191-01PT(D2)	62.7	Female	T2D	10	nPOD	19.9	8.1	52.7

7	IG62_6059-06PT(D2)	18.8	Female	T2D	0.3	nPOD	39.3	6.1	18.6
8	IG63_6110-05PT(D2)	20.7	Female	T2D	0	nPOD	40	5.9	20.7

HLA and autoantibody status

	Patient	Autoantibody Status	HLA Risk	HLA-A	HLA-B	HLA-DRB1	HLA-DQB1	HbA1c	HbA1c (mmol/mol)
1	IG1_6013-04PT(C)	Negative	Neutral	*01:01/01:01	*07:02/44:03	*01:02/13:01	*05:01/06:03	0	-
2	IG2_6024-01PT(C)	Negative	Predisposing	*01:01/31:01	*07:02/08:01	*03:01/13:02	*02:01/06:04	0	-
3	IG3_6048-04PT(C)	Negative	Protective	*03:01/31:01	*07:02/47:01	*04:01/15:01	*03:01/06:02	0	-
4	IG4_6075-06PB(C)	Negative	Neutral	*23:01/33:03	*15:03/58:01	*07:01/11:01	*02:01/03:01	0	-
5	IG5_6012-02PT(C)	Negative	Protective	*02:01/02:01	*07:02/57:01	*07:01/15:01	*03:03/06:02	0	-
6	IG6_6099-02PB(C)	Negative	Predisposing	*24:02/26:01	*27:03/44:05	*03:01/11:01	*02:01/03:01	0	-
7	IG7_6140-04PT(C)	Negative	Predisposing	*01:01/03:01	*35:01/35:01	*01:01/03:01	*02:01/05:01	6	42.1
8	IG8_6162-01PT(C)	Negative	Neutral	* 25:01/68:02	NA	* 13:03/16:02	* 03:01/05:02	0	-
9	IG9_6168-04PT(C)	Not tested	Predisposing	*02:01/24:02	NA	*01:03/04:04	*03:02/05:01	6.2	44.3
10	IG10_6227-02PT(C)	Negative	Predisposing	*02:01/03:01	NA	*04:01/13:02	*03:02/06:04	0	-
11	IG11_6129-02PT(C)	Negative	Protective	*03:01/23:01	*07:02/14:02	*03:01/15:01	*02:01/06:02	5.2	33.3
12	IG12_6165-04PT(C)	Negative	Protective	*01:01/02:01	NA	*13:01/15:01	*06:02/06:03	5.6	37.7
13	IG13_6102-04PT(C)	Negative	Predisposing	*03:01/31:01	*08:01/44:02	*03:01/04:01	*02:01/03:01	6.1	43.2
14	IG14_6229-02PT(C)	Negative	Neutral	*02:01/24:02	NA	*01:01/13:02	*05:04/06:04	5.5	36.6
15	IG15_6251-02PT(C)	Negative	Neutral	*02:01/24:02	NA	*04:01/11:01	*03:01/03:01	5.3	34.4
16	IG16_6010-04PT(C)	Not tested	Protective	* 02:01/02:01	* 07:02/57:01	* 07:01/15:01	* 03:03/06:02	0	-
17	IG17_6179-04B PB(C)	Negative	Predisposing	*02:01/24:02	NA	*03:01/04:04	*02:01/03:02	0	-
18	IG18_6019-04PT(C)	Negative	Predisposing	*faint. faint	*faint. faint	*01:01/03:01	*02:01/05:01	5.6	37.7
1	IG40_6080-01PT(AB)	GADA+/mIAA	Neutral	*02:01/11:01	*35:01/44:02	*01:01/04:01	*03:01/05:01	0	-
2	IG41_6123-02PT(AB)	GADA+	Neutral	*02:01/24:02	*35:01/51:01	*08:01/11:01	*03:01/04:02	5.4	35.5
3	IG42_6158-10PT(AB)	GADA+/IA2A+	Neutral	*03:01/24:02	*15:01/49:01	*04:01/13:02	*03:01/06:04	5.6	37.7
4	IG43_6167-08PT(AB)	IA2A+/ZnT8A+	Predisposing	*01:01/03:01	NA	*04:04/15:02	*03:02/06:01	0	-
5	IG44_6171-01PT(AB)	GADA+	Predisposing	*02:01/31:01	NA	*03:01/03:01	*02:01/02:01	0	-
6	IG45_6044-02PT(AB)	IA2A+	Protective	*01:01/33:01	*14:02/37:01	*01:02/15:01	*05:01/06:02	0	-
7	IG46_6101-04PT(AB)	GADA+	Protective	*02:01/32:01	*44:02/51:01	*13:02/15:01	*06:02/06:04	0	-
8	IG47_6154-06PT(AB)	GADA+	Protective	*02:01/03:01	*07:02/07:02	*09:01/15:01	*03:03/06:03	0	-
9	IG48_6156-06PT(AB)	GADA+	Neutral	*03:01/32:01	*40:02/53:01	*01:01/01:02	*05:01/05:01	0	-
10	IG49_6181-06APT(AB)	GADA+	Predisposing	*03:01/11:01	NA	*01:01/04:01	*03:02/05:01	0	-
11	IG50_6197-08PB(AB)	GADA+/IA2A+	Neutral	*02:02/24:02	NA	*03:02/07:01	*02:02/04:02	5.5	36.6

12	IG51_6147-02PB(AB)	GADA+	Neutral	*02:01/02:01	*08:01/15:01	*03:01/04:01	*03:01/04:02	5.2	33.3
1	IG64_DiViD-6(Dg)	GAD		*0101.*2902	*0801.*4403	*0301.*0701	*0201.*0202	7.1	54.1
2	IG65_DiViD-5(Dg)	GAD/INS/IA2		*0201.*0301	*1801.*4001	*0301.*0401	*0201.*0302	7.4	57.4
3	IG66_DiViD-4(Dg)	GAD/INS/IA2		*0201	*1501.*3501	*0401	*0302	7.4	57.4
4	IG67_DiViD-3(Dg)	GAD/ZnT8/IA2		*0101.*2402	*0801.*1501	*0301.*0401	*0201.*0302	7.1	54.1
5	IG68_DiViD-2(Dg)	GAD/ZnT8/IA2		*0201.*1101	*1801.*4001	*0401.*1301	*0302.*0603	10.3	89.1
1	IG20_6070-06PT(D1)	IA2A+/mIAA	Neutral	*02:01/02:05	*38:01/58:01	*10:01/16:01	*05:01/05:02	0	-
2	IG21_6084-02PT(D1)	mIAA+	Predisposing	*01:01/32:01	*08:01/40:01	*03:01/04:01	*02:01/03:02	0	-
3	IG22_6088-02PH(D1)	GADA+/IA2A+/ZnT8A+/mIAA+	Predisposing	*02:01/33:01	*14:02/51:01	*01:01/03:01	*02:01/05:01	0	-
4	IG23_6180-04PT(D1)	GADA+/IA2A+/ZnT8A+/mIAA+	Predisposing	*01:01/02:01	NA	*01:01/03:01	*02:01/05:01	0	-
5	IG24_6224-01PT(D1)	Negative	Predisposing	*02:01/11:01	NA	*01:01/04:04	*03:02/05:01	0	-
6	IG25_6243-02PT(D1)	mIAA+	Predisposing	*01:01/02:01	NA	*03:01/03:01	*02:01/02:01	13.1	119.7
7	IG26_6228-04PT(D1)	GADA+/IA2A+/ZnT8A+	Predisposing	*23:01/68:01	NA	*03:01/04:02	*02:01/03:02	13.3	121.9
8	IG27_6209-01PT(D1)	IA2A+/ZnT8A+/mIAA	Predisposing	*01:01/02:01	NA	*04:01/03:01	*03:02/02:01	0	-
9	IG28_6038-06PH(D1)	Negative	Predisposing	*02:01/03:01	*35:01/44:05	*01:01/03:01	*02:01/05:01	0	-
10	IG29_6046-03PT(D1)	IA2A+/ZnT8A+	Predisposing	*02:01/03:01	*15:01/39:01	*01:01/04:01	*03:02/05:01	0	-
11	IG30_6069-06PT(D1)	Not tested	Predisposing	*02:01/02:01	*51:02/58:01	*03:01/12:02	*02:01/03:01	0	-
12	IG31_6195-06PT(D1)	GADA+/IA2A+/ZnT8A+/mIAA+	Neutral	*03:01/03:01	NA	*04:01/15:01	*03:02/06:02	0	-
13	IG32_6052-03PB(D1)	IA2A+/mIAA+	Neutral	*02:01/68:01	*27:05/50:01	*09:01/16:02		0	-
14	IG33_6268-02PT(D1)	mIAA+	Predisposing	*02:01/68:01	NA	*03:01/13:02	*02:01/06:04	9.8	83.6
15	IG34_6265-02PH(D1)	GADA+/mIAA+	Predisposing	*03:01/32:01	NA	*03:01/04:01	*02:01/03:02	0	-
16	IG35_6196-04PT(D1)	GADA+/mIAA+	Neutral	*03:01/30:02	NA	*12:01/13:03	*02:01/05:01	0	-
17	IG36_6211-04PT(D1)	GADA+/IA2A+/ZnT8A+/mIAA+	Predisposing	*02:01/03:01	NA	*04:05/12:01	*03:01/03:02	10.5	91.3
18	IG37_6113-01PH(D1)	mIAA+	Predisposing	*02:01/03:01	*08:01/44:02	*01:01/03:01	*02:01/05:01	0	-
19	IG38_6264-02PB(D1)	Negative	Predisposing	*23:01/32:01	NA	*03:01/04:04	*02:01/03:02	8.9	73.8
20	IG39_6135-01PT(D1)	mIAA+	Neutral	*02:01/68:01	*27:05/51:01	*01:01/13:02	*05:01/06:04	0	-
1	IG56_6188-06PT(D2)	Negative	Neutral	*02:02/66:01	NA	*12:01/14:01	*03:01/06:04	7.2	55.2
2	IG57_6114-03PT(D2)	Negative	Protective	*03:01/31:01	*07:02/15:01	*07:01/15:01	*03:03/06:02	7.8	61.7
3	IG58_6249-01PT(D2)	mIAA+	Predisposing	*02:01/24:02	NA	*04:05/04:05	*04:02/04:02	0	-
4	IG59_6275-02PT(D2)	Negative	Protective	*02:01/25:01	NA	*09:01/15:01	*03:03/06:02	0	-
5	IG60_6273-04PT(D2)	Negative	Protective	*02:02/03:01	NA	*15:01/15:03	*06:02/06:02	0	-
6	IG61_6191-01PT(D2)	Negative	Predisposing	*01:01/02:01	NA	*04:01/07:01	*02:02/03:02	6	42.1
7	IG62_6059-06PT(D2)	Negative	Neutral	*02:01/11:01	*35:01/50:01	*07:01/08:02	*02:01/04:02	0	-
8	IG63_6110-05PT(D2)	Negative	Neutral	*23:01/34:02	*14:02/53:01	*03:02/15:03	*04:02/06:02	0	-

ESM Table 2: SNPs associated with T1D. First three columns show SNP number, chromosome placement, and position. Both the p value without Q-value filtering (column four) and with eQTL p values were shown (last three columns). [a] Indicate genes that also showed dysregulation on RNA expression level. [b] Newly discovered in association with T1D.

SNP	CHR	POS(GRCh38)	T1D ImmunoChip P-value	OR (MinAllele)	Maj>Min allele	Associated Gene	RegulomeDb Rank	Validated e-QTLs: Whole blood p-value	Predicted e-OTLs: Pancreas p value	Predicted e-OTLs: Whole blood p value
rs4845618	chr1	154427539	1.79E-02	1.046	T>G	ADAR[a,b]	4			
rs7529229	chr1	154448302	1.79E-02	0.956	T>C	ADAR[a,b]	2b			
rs7553796	chr1	154431930	8.50E-03	1.0513	C>A	ADAR[a,b]	2b			
rs2228145	chr1	154454494	8.50E-03	0.9512	A>C	ADAR[a,b]	4			
rs4845371	chr1	154435864	7.27E-03	1.0523	C>T	ADAR[a,b]	4			
rs6667434	chr1	154436624	7.27E-03	1.0523	G>A	ADAR[a,b]	5			
rs4129267	chr1	154453788	6.20E-03	0.9493	C>T	ADAR[a,b]	2b			
rs4845625	chr1	154449591	1.32E-03	1.0629	C>T	ADAR[a,b]	5			0.019
rs2229238	chr1	154465420	4.37E-05	1.0986	C>T	ADAR[a,b]	4			
rs6827840	chr4	184423778	4.61E-03	1.0887	C>T	CASP3	7			
rs17075783[b]	chr4	184404338	4.46E-03	0.913	T>C	CASP3	3a		0.028	
rs2968482	chr16	88778025	1.88E-02	1.0481	T>C	CYBA	2b			
rs9788969	chr16	88745842	1.79E-02	0.956	T>C	CYBA	2b			
rs3826076	chr16	88714110	1.08E-02	0.924	C>T	CYBA	2b			
rs4782429[b]	chr16	88725689	4.21E-03	0.9204	G>A	CYBA	5			
rs2372914	chr2	37037532	1.96E-02	0.9589	T>C	EIF2AK2	6	4.59E-5		
rs4233921	chr2	37127006	1.43E-02	0.9522	C>T	EIF2AK2	7	8.32E-11		
rs6734118	chr2	37332212	1.24E-02	1.0565	C>A	EIF2AK2	6	3.9E-8		0.00017
rs2247010[b]	chr2	36984959	3.73E-03	0.9436	A>G	EIF2AK2	7	6.01E-7		
rs1446534[b]	chr8	116944800	1.69E-04	0.924	G>A	EIF3H	7			0.0064
rs9927281	chr16	11659567	1.90E-02	0.9343	G>A	GSPT1	2b			
rs350234[b]	chr16	12100497	1.64E-02	1.0492	A>G	GSPT1	4			
rs17710179	chr2	162112761	1.93E-02	1.1241	A>G	IFIH1[a]	2b			
rs79820653	chr2	162154167	1.70E-02	1.0768	C>A	IFIH1[a]	5			
rs74387251	chr2	162167034	1.62E-02	0.7603	A>C	IFIH1[a]	7			
rs7599142	chr2	162136981	1.55E-02	0.955	C>T	IFIH1[a]	6			
rs2080712	chr2	162105419	1.45E-02	1.045	G>A	IFIH1[a]	6			
rs77506733	chr2	162445728	1.37E-02	1.148	T>C	IFIH1[a]	7			
rs35744605	chr2	162277580	1.34E-02	0.7196	G>T	IFIH1[a]	4			
rs13022784	chr2	162480693	1.30E-02	1.0747	A>G	IFIH1[a]	6			
rs77446212	chr2	162404017	1.29E-02	1.0964	T>G	IFIH1[a]	3a			
rs16846296	chr2	162109592	1.21E-02	1.1366	A>G	IFIH1[a]	7			
rs73973006	chr2	162141100	1.10E-02	1.1298	?/?	IFIH1[a]	7			
rs142735009	chr2	162345514	1.00E-02	0.7749	?/?	IFIH1[a]	3a			
rs77088072	chr2	162294461	9.94E-03	0.7929	G>A	IFIH1[a]	5		0.0014	0.0043
rs11885343	chr2	162112363	8.50E-03	0.9512	C>T	IFIH1[a]	3a			
rs11897585	chr2	162113152	8.50E-03	0.9512	A>G	IFIH1[a]	2b			
rs11886859	chr2	162113193	8.50E-03	0.9512	C>G	IFIH1[a]	4			
rs10185053	chr2	162115829	8.50E-03	0.9512	A>G	IFIH1[a]	5			
rs10185151	chr2	162115875	8.50E-03	0.9512	A>G	IFIH1[a]	5			
rs12052431	chr2	162118887	8.50E-03	0.9512	C>T	IFIH1[a]	4			
rs10174693	chr2	162119581	8.50E-03	0.9512	C>A	IFIH1[a]	7			
rs17762054	chr2	162154176	8.46E-03	1.1712	T>C	IFIH1[a]	5			

rs2892826	chr2	162111748	7.27E-03	0.9503	C>A	IFIH1[a]	5			
rs6432709	chr2	162117382	7.27E-03	0.9503	A>C	IFIH1[a]	6			
rs10171070	chr2	162118368	7.27E-03	0.9503	C>T	IFIH1[a]	4			
rs2075300	chr2	162118466	7.27E-03	0.9503	T>C	IFIH1[a]	3a			
rs10180049	chr2	162136962	7.27E-03	0.9503	G>A	IFIH1[a]	6			
rs10209700	chr2	162138649	7.27E-03	0.9503	A>G	IFIH1[a]	7			
rs13422273	chr2	162273547	6.85E-03	0.7672	?/?	IFIH1[a]	6			
rs888274	chr2	162481139	6.39E-03	0.9418	C>T	IFIH1[a]	5		0.033	
rs2241242	chr2	162118384	6.20E-03	0.9493	G>A	IFIH1[a]	4			
rs10171915	chr2	162118993	6.20E-03	0.9493	G>T	IFIH1[a]	2b			
rs2389681	chr2	162134737	6.20E-03	0.9493	G>T	IFIH1[a]	7			
rs2111489	chr2	162135165	6.20E-03	0.9493	T>G	IFIH1[a]	6			
rs1558862	chr2	162135374	6.20E-03	0.9493	A>T	IFIH1[a]	6			
rs2241243	chr2	162118677	5.28E-03	0.9484	G>A	IFIH1[a]	2b			
rs12692647	chr2	162122301	5.28E-03	0.9484	C>T	IFIH1[a]	7			
rs10930043	chr2	162123411	5.28E-03	0.9484	A>G	IFIH1[a]	5			
rs10490423	chr2	162125936	5.28E-03	0.9484	C>T	IFIH1[a]	5			
rs35596334	chr2	162132318	5.28E-03	0.9484	C>T	IFIH1[a]	7			
rs2389680	chr2	162133393	5.28E-03	0.9484	A>G	IFIH1[a]	6			
rs2216338	chr2	162133458	5.28E-03	0.9484	G>A	IFIH1[a]	7			
rs7564122	chr2	162134217	5.28E-03	0.9484	A>G	IFIH1[a]	7			
rs79475499	chr2	162192461	4.98E-03	1.1411	C>A	IFIH1[a]	5			
rs34382064	chr2	162384634	4.65E-03	1.1618	C>A	IFIH1[a]	5			
rs62189714	chr2	162110331	4.48E-03	0.9474	C>A	IFIH1[a]	5			
rs10198168	chr2	162119481	4.48E-03	0.9474	A>T	IFIH1[a]	7			
rs7607713	chr2	162133780	4.48E-03	0.9474	G>A	IFIH1[a]	7			
rs7568666	chr2	162133859	4.48E-03	0.9474	T>C	IFIH1[a]	7			
rs80063898	chr2	162164613	4.36E-03	1.1434	C>G	IFIH1[a]	7			
rs2193495	chr2	162125628	3.79E-03	0.9465	T>C	IFIH1[a]	6			
rs7598422	chr2	162136221	3.20E-03	0.9455	G>T	IFIH1[a]	7			
rs77693762	chr2	162422765	2.23E-03	0.8985	C>T	IFIH1[a]	5			
rs62188206	chr2	162488054	2.15E-03	0.9148	G>T	IFIH1[a]	5			
rs75166367	chr2	162107791	1.90E-03	1.1252	G>A	IFIH1[a]	4			
rs17728270	chr2	162440681	1.75E-03	1.1019	T>C	IFIH1[a]	7			
rs977907	chr2	162221345	1.71E-03	1.148	T>A	IFIH1[a]	4			
rs71424730	chr2	162184894	1.45E-03	1.1286	A>G	IFIH1[a]	3a			
rs34298676	chr2	162269807	1.43E-03	1.1252	A>G	IFIH1[a]	7			
rs35732034	chr2	162268086	1.37E-03	0.7054	C>T	IFIH1[a]	7			
rs17715343	chr2	162311236	1.22E-03	0.8958	C>G	IFIH1[a]	7			
rs17713557	chr2	162260675	9.76E-04	1.1298	G>T	IFIH1[a]	5			
rs35701596	chr2	162232496	8.04E-04	1.132	G>T	IFIH1[a]	6			
rs5649	chr2	162147348	7.73E-04	0.6288	C>T	IFIH1[a]	5			
rs114779135	chr2	162120286	6.96E-04	1.1688	A>C	IFIH1[a]	4			
rs71424731	chr2	162218378	6.61E-04	1.1343	T>C	IFIH1[a]	6			
rs145769275	chr2	162251238	4.77E-04	0.7695	?/?	IFIH1[a]	7			
rs35696140	chr2	162469314	2.09E-04	0.9148	A>G	IFIH1[a]	4			
rs41368446	chr2	162141603	1.82E-04	1.14	T>G	IFIH1[a]	6			
rs13416088	chr2	162141149	1.07E-04	0.9112	G>A	IFIH1[a]	7			
rs3764917	chr2	162173826	8.98E-05	0.9103	T>C	IFIH1[a]	6			
rs72871627	chr2	162280432	8.21E-05	0.6825	A>G	IFIH1[a]	6			
rs17716942	chr2	162404181	3.43E-05	0.8905	T>C	IFIH1[a]	4			
rs13432094	chr2	162339378	3.21E-05	0.8538	A>T	IFIH1[a]	7			
rs76684000	chr2	162445184	6.07E-06	0.827	?/?	IFIH1[a]	7			

rs114165927	chr2	162261394	5.02E-06	0.8369	G>C	IFIH1[a]	2b			
rs13422767	chr2	162243749	5.33E-08	0.8728	G>A	IFIH1[a]	7		0.015	
rs13022749	chr2	162415697	1.60E-08	0.8932	T>A	IFIH1[a]	5			0.0051
rs2075302	chr2	162219636	1.46E-08	1.1074	T>C	IFIH1[a]	5			
rs11679244	chr2	162225885	1.05E-08	1.1085	C>A	IFIH1[a]	6			
rs3788964	chr2	162350970	3.99E-09	0.8581	T>C	IFIH1[a]	7		0.028	
rs7587426	chr2	162353833	5.28E-10	0.8887	T>C	IFIH1[a]	7			
rs7608315	chr2	162357213	9.56E-11	0.8843	A>C	IFIH1[a]	7			0.025
rs3747517	chr2	162272314	4.98E-11	0.8711	C>T	IFIH1[a]	7		0.035	0.022
rs35667974	chr2	162268127	1.01E-11	0.5724	?/?	IFIH1[a]	7			
rs1549020	chr2	162384337	2.56E-12	0.8633	T>G	IFIH1[a]	6			0.0048
rs13023380	chr2	162297853	2.56E-12	0.8755	A>G	IFIH1[a]	3a			0.046
rs2068330	chr2	162380880	3.64E-16	0.8496	C>G	IFIH1[a]	6			0.023
rs984971	chr2	162368011	2.40E-16	0.8487	A>G	IFIH1[a]	7			0.025
rs1990760	chr2	162267541	5.84E-17	0.853	T>C	IFIH1[a]	7			
rs2111485	chr2	162254026	3.81E-18	0.8479	G>A	IFIH1[a]	7			
rs3993797	chr11	511580	1.64E-02	0.8869	C>T	IFITM1	4			
rs2834136[b]	chr21	33200486	1.36E-02	0.8538	G>A	IFNAR1[a]	6			
rs2834136[b]	chr21	33200486	1.36E-02	0.8538	G>A	IFNAR2	6			
rs7316231	chr12	68075862	1.79E-02	1.046	A>G	IFNG[a]	5	0.0015		
rs4913395	chr12	68076705	1.79E-02	1.046	T>C	IFNG[a]	6	0.0023		
rs11614178	chr12	68114342	1.55E-02	0.955	G>A	IFNG[a]	7	2.02E-4		
rs7132610[b]	chr12	68114875	3.18E-03	1.1252	T>A	IFNG[a]	5			
rs11123914	chr2	102230272	1.30E-02	0.9259	?/?	IL1R1	5		7.60E-08	
rs1558647	chr2	102193725	7.42E-03	0.9204	?/?	IL1R1	5		1.60E-07	
rs1922294	chr2	102219449	7.42E-03	0.9204	?/?	IL1R1	7		1.60E-07	
rs6731416[b]	chr2	101911513	6.70E-03	0.7543	T>C	IL1R1	7			
rs7565504[b]	chr2	101917865	6.70E-03	0.7543	A>G	IL1R1	5			
rs140672656[b]	chr2	101918850	6.33E-03	0.7843	?/?	IL1R1	6			
rs79184888[b]	chr2	101955759	5.83E-03	0.7528	G>A	IL1R1	7			
rs4850992[b]	chr2	101987400	5.58E-03	0.7475	A>G	IL1R1	4			
rs3993797[b]	chr11	511580	1.64E-02	0.8869	C>T	IRF7[a]				
rs17758[b]	chr11	644325	1.01E-02	0.8659	G>A	IRF7[a]	4			
rs118067545[b]	chr11	682373	8.48E-03	0.8361	C>T	IRF7[a]	5			
rs7278439	chr21	41606174	1.24E-02	0.9512	C>T	MX1[a]	4			
rs2838069	chr21	41598049	1.15E-02	0.9531	C>A	MX1[a]	5			
rs7278439[b]	chr21	41606174	1.24E-02	0.9512	C>T	MX2[a,b]	4		0.012	
rs2838069	chr21	41598049	1.15E-02	0.9531	C>A	MX2[a,b]	5			
rs2066843	chr16	50711288	1.96E-02	1.0502	C>T	NOD2	5			1.70E-12
rs17313265	chr16	50713793	1.96E-02	1.0502	C>T	NOD2	3a			2.40E-12
rs1548914	chr16	50445460	8.91E-03	0.9343	A>C	NOD2	5			
rs2302759[b]	chr16	50793690	4.11E-03	0.9361	G>A	NOD2	5		0.014	4.40E-07
rs1034687[b]	chr12	112732362	2.16E-07	0.8694	G>A	OAS1[a]	7	2.19E-11		
rs1015249[b]	chr12	112687506	1.25E-07	0.8949	C>A	OAS1[a]	5	2.7E-13		
rs4767000[b]	chr12	112730563	1.03E-09	1.123	G>A	OAS1[a]	7			
rs739744[b]	chr12	112747657	1.03E-09	1.123	A>G	OAS1[a]	7	1.11E-21		
rs1034687[b]	chr12	112732362	2.16E-07	0.8694	G>A	OAS2[a]	7			
rs4767000[b]	chr12	112730563	1.03E-09	1.123	G>A	OAS2[a]	7			
rs739744[b]	chr12	112747657	1.03E-09	1.123	A>G	OAS2[a]	7			
rs1034687[b]	chr12	112732362	2.16E-07	0.8694	G>A	OAS3[a]	7		0.013	
rs4767000[b]	chr12	112730563	1.03E-09	1.123	G>A	OAS3[a]	7			
rs739744[b]	chr12	112747657	1.03E-09	1.123	A>G	OAS3[a]	7	1.32E-4		
rs17125267[b]	chr12	53277466	1.03E-02	0.8378	C>T	SP1	5			

rs11170466	chr12	53192075	1.47E-05	1.1841	G>A	SP1	4			
rs2286596	chr12	6443889	1.64E-02	0.9531	A>G	TNFRSF1A	1d			
rs61311839	chr12	6413035	1.15E-02	0.9531	?/?	TNFRSF1A	5			0.048
rs4764589	chr12	6423116	8.50E-03	0.9512	?/?	TNFRSF1A	5			
rs4764590	chr12	6423184	8.50E-03	0.9512	?/?	TNFRSF1A	7			
rs749930	chr12	6311347	7.10E-03	1.0725	C>T	TNFRSF1A	4			
rs2250430	chr12	6312008	4.37E-03	1.0587	T>A	TNFRSF1A	4			
rs7300535	chr12	6410971	2.70E-03	0.9418	?/?	TNFRSF1A	6			
rs11064182	chr12	6418071	2.70E-03	0.9446	?/?	TNFRSF1A	5			
rs7961280	chr12	6418903	2.70E-03	0.9446	?/?	TNFRSF1A	4			
rs11064180	chr12	6414083	2.27E-03	0.9436	?/?	TNFRSF1A	5			
rs12812284	chr12	6417451	1.90E-03	0.9427	?/?	TNFRSF1A	5			
rs10849451[b]	chr12	6415513	1.59E-03	1.0618	?/?	TNFRSF1A	6			0.028
rs10849453	chr12	6419086	1.10E-03	0.9399	?/?	TNFRSF1A	5			
rs2364482	chr12	6392965	6.96E-04	0.925	T>G	TNFRSF1A	3a			
rs12296430	chr12	6394334	5.44E-04	0.9204	?/?	TNFRSF1A	4			
rs11671087	chr19	39271150	6.39E-03	0.9418	T>C	ZFP36	5			
rs7359953	chr19	39273073	6.39E-03	0.9418	G>A	ZFP36	5			
rs7359950	chr19	39273125	6.39E-03	0.9418	C>T	ZFP36	5			

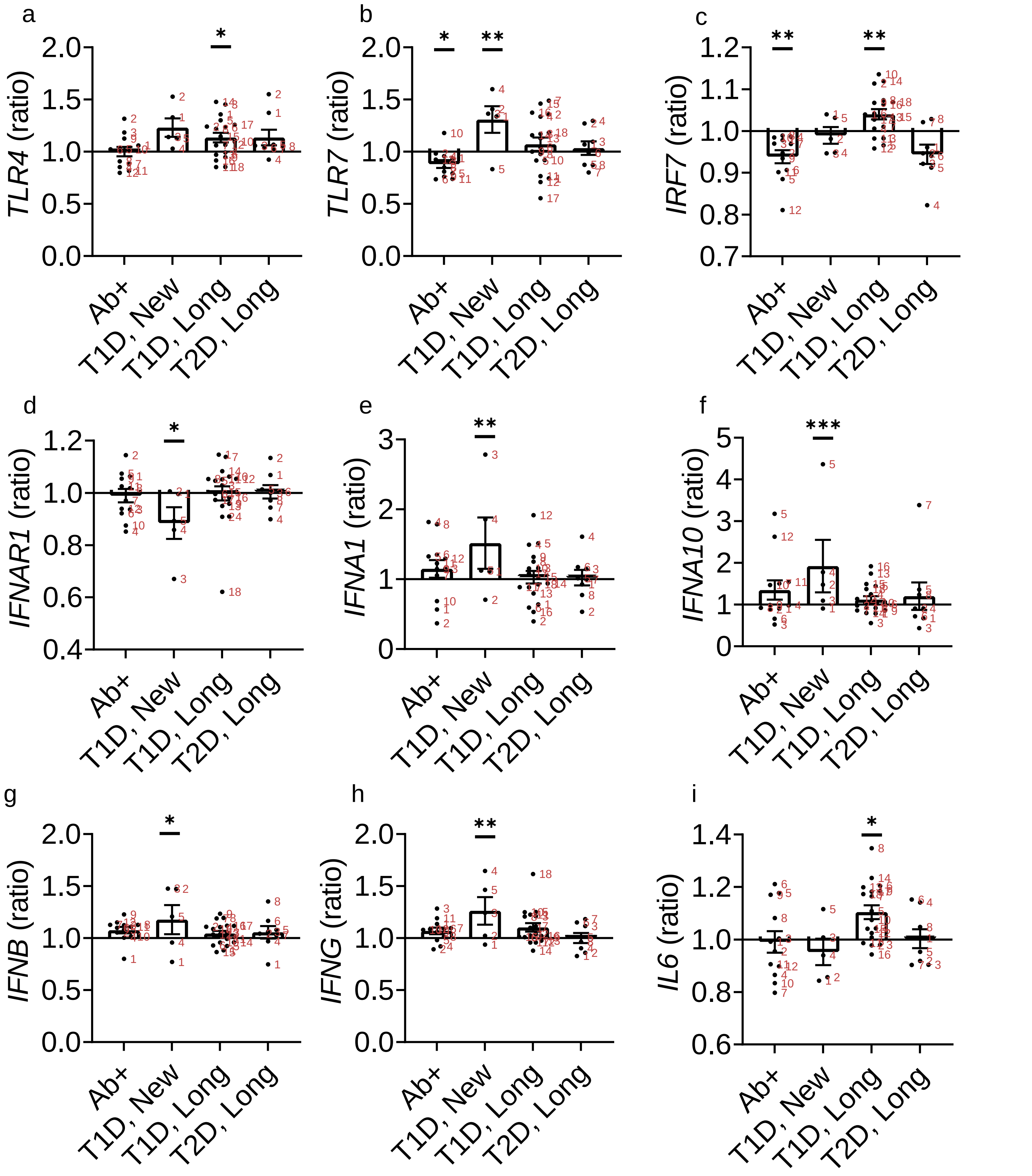

ESM Fig. 1 Genes involved in PAMP recognition and IFN induction. The RNA expression levels of TLR4 (a), TLR7 (b), IRF7 (c), IFNAR1 (d), IFNA1 (e), IFNA10 (f), IFNB (g), IFNG (h) and IL6 (i) were transformed, and a mean was calculated. The ratio between the mean expression level in each group (autoantibody-positive [Ab+], new-onset type 1 diabetes [T1D, New], longstanding type 1 diabetes [T1D, Long], longstanding type 2 diabetes [T2D, Long]) and the non-diabetic control group was graphed. *p<0.05, **p<0.01 and ***p<0.001 vs control group. Each data point is numbered for comparison with demographic features, HLA and autantibody status in ESM Table 2.

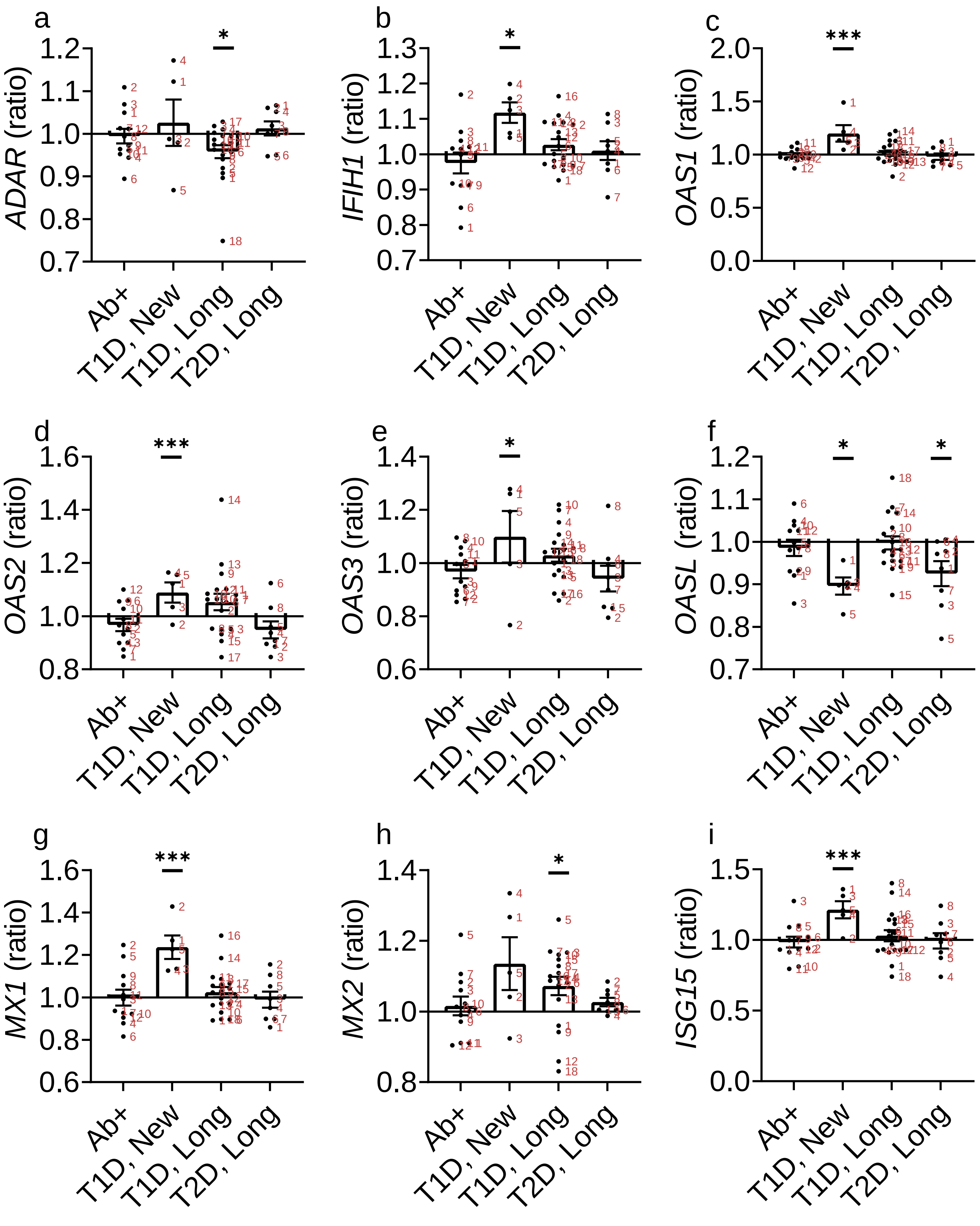

ESM Fig. 2 Cytokine genes and ISGs that were upregulated by IFN induction. The RNA expression levels of ADAR (a), IFIH1 (b), OAS1 (c), OAS2 (d), OAS3 (e), OASL (f), MX1 (g), MX2 (h) and ISG15 (i) were transformed, and a mean was calculated. The ratio between the mean expression level in each group (autoantibody-positive [Ab+], new-onset type 1 diabetes [T1D, New], longstanding type 1 diabetes [T1D, Long], longstanding type 2 diabetes [T2D, Long]) and the non-diabetic control group was graphed. *p<0.05 and ***p<0.001 vs control group. Each data point is numbered for comparison with demographic features, HLA and autantibody status in ESM Table 2.
